# Activation of Nrf2-mediated anti-oxidant genes by antrodin C prevents hyperglycemia-induced senescence and apoptosis in human endothelial cells

**DOI:** 10.18632/oncotarget.19951

**Published:** 2017-08-04

**Authors:** Kumar K.J. Senthil, Vani M. Gokila, Sheng-Yang Wang

**Affiliations:** ^1^ Department of Forestry, National Chung Hsing University, Taichung, Taiwan; ^2^ National Chung Hsing University/University of California at Davis, Plant and Food Biotechnology Center, National Chung Hsing University, Taichung, Taiwan; ^3^ Agricultural Biotechnology Research Center, Academia Sinica, Taipei, Taiwan

**Keywords:** antrodin C, Antrodia cinnamomea, high glucose, endothelial cells, senescence, Gerotarget

## Abstract

In the present study, we investigated the effects of antrodin C (ADC), a maleimide derivative isolated from mycelia of *Antrodia cinnamomea*, on high glucose (HG, 30 mM)-accelerated endothelial dysfunction *in vitro*. HG-induced cytotoxicity in human umbilical vein endothelial cells (HUVECs) was significantly ameliorated by ADC. In addition, treatment with ADC significantly prevented HG-induced senescence, growth arrest at the G_1_-S transition phase and apoptosis in HUVECs. Moreover, the increased level of intracellular reactive oxygen species (ROS) under HG condition was significantly ameliorated by ADC. Further analysis revealed that ADC-mediated anti-oxidant effects were due to up-regulation of cellular anti-oxidant genes, such as HO-1 and NQO-1 *via* promotion of the transcriptional activity of Nrf2, which was further confirmed by the failure of ADC to protect HUVECs from HG-induced dysfunction under HO-1 inhibition or Nrf2 silencing. Furthermore, hyperosmotic glucose (HOG, 60 mM)-induced uncontrolled production of ROS, rapid apoptotic cell death and HUVEC injury were significantly prevented by ADC, whereas these preventive effects were barely observed in HO-1 inhibited or Nrf2 silenced cells. Taken together, these results suggest that ADC may represent a promising intervention in diabetic-associated cardiovascular diseases by activating the Nrf2-dependent cellular anti-oxidant defense system.

## INTRODUCTION

Hyperglycemia, a characteristic feature of diabetes mellitus (DM) and metabolic syndrome has emerged as a major health problem that rapidly causes vascular and organ dysfunction [1]. It has been estimated that by 2030, developing countries in Asia and the Middle East will have the largest increases in the prevalence of type-2 DM due to modernization of lifestyles and nutrition [2]. Common complications of diabetes mellitus include age-associated diseases, such as vascular ageing, hypertension and atherosclerosis [3]. Vascular endothelial cells are highly specialized and active cells that regulate thrombosis and inflammatory processes. Thus, alterations in *endothelial* cells and the vasculature play a critical role in the pathogenesis of a broad spectrum of the most serious human diseases [4]. Vascular endothelial cell senescence, which is highly associated with diabetes mellitus [5], promotes vascular dysfunction and is accompanied by increased vascular risk [6].

Vascular senescence can be induced by a plethora of internal or external insults, including telomere dysfunction [7], ionizing radiation [8], reactive oxygen species (ROS) [9], inflammatory cytokines [10, 11], drugs [12] and high glucose [13, 14]. Increasing evidence indicates that high glucose, a characteristic feature of diabetes mellitus, induces oxidative stress, which invokes irreversible growth arrest *in vitro* within a few days, a term referred to as stress-induced premature senescence [15]. It has been established that hyperglycemia-induced cell-cycle arrest in endothelial cells is mediated by p21^CIP1^ and p16^INK4A^, two cyclin-dependent kinase inhibitors (CDKs) [16]. In addition, previous studies have demonstrated that exposure of vascular endothelial cells to high glucose causes a significant increase in apoptosis, possibly associated with an increase in intracellular ROS, alteration in fatty-acid metabolism, impaired Akt activation by insulin and increased caspase-3 activity [17, 18]. In the pathological state, oxidative stress results in excessive production of ROS. ROS, include free radicals such as superoxide and hydroxyl radicals, and non-radical species (hydrogen peroxide). Excessive ROS generation overwhelms endogenous antioxidant systems, and overproduction of ROS also reduces the efficacy of endogenous antioxidants. Under such conditions, induction of antioxidants by external factors plays a critical role in cellular stress response [19, 20].

Eukaryotic cells have a primary and secondary defense mechanism to respond to oxidative stresses. In particular, phase I enzymes such as cytochrome p450 and phase II enzymes, including heme oxygenase-1 (HO-1), NAD(P) H:quinone oxidoreductase 1 (NQO1) and glutathione-*S*-transferase (GST) are rapidly activated by an endogenous mechanism through which oxidative toxicants are removed before they can damage the DNA [21]. Transcriptional activation of antioxidants or detoxifying genes are predominantly regulated by a redox-sensitive transcription factor nuclear factor erythroid 2-related factor-2 (Nrf2) [22]. Dietary phytochemicals are identified as potent activators of cellular antioxidant genes through the induction of the Nrf2 signaling pathway thereby bolstering the anti-oxidant defense system in a variety of cells.

*Antrodia cinnamomea* (Syn. *Antrodia camphorate* or *Taiwanofungus camphoratus*) is a unique medicinal mushroom that has long been used as a Chinese folk medicine in Taiwan for the treatment of various human illnesses including, liver diseases, food and drug intoxication, diarrhea, abdominal pain, hypertension, allergies, skin and tumorigenic diseases [23-25]. Recent scientific investigations have revealed that *A. cinnamomea* has extensive pharmacological effects including anti-cancer, anti-inflammation, anti-oxidant, anti-microbial, anti-diabetic, anti-hypertensive, anti-hyperlipidemia, anti-metastasis, immunomodulatory, hepatoprotective and neuroprotective effects [23-25]. The therapeutic efficacy of this mushroom may be due its high phytocompound content which includes terpenoids, polysaccharides, benzenoids, lignans, nucleic acid, benzoquinone derivatives, steroids, and maleic/succinic acid derivatives. In addition, *A. cinnamonea* is one of the richest sources of biologically active compounds such as antcins, anticinates, antrodins and antroquinonls [25].

Initially, Nakamura et al. [26] isolated 5 new maleic and succinic acid derivatives from the mycelia of *A. cinnamomea*, and among them, the maleimide derivatives antrodin B (ADB) and antrodin C (ADC) exhibited anti-cancer properties in Lewis lung carcinoma cells *in vitro*. Subsequent studies also demonstrated that the pyrrolidione ADC strongly inhibited the lipopolysaccharide (LPS)-induced production of pro-inflammatory cytokines and chemokines in macrophage cells [27, 28]. Recently, we reported that ADC possesses anti-metastatic effects in human breast cancer cells through the inhibition of epithelial-to-mesenchymal transition and tumor cell migration *in vitro* [29]. However, other biological effects of this pharmacologically important compound are largely unknown. In this study, the protective effects of ADC on hyperglycemia-induced vascular endothelial cell senescence and apoptosis were examined. The anti-oxidant potential of ADC was compared with the known anti-oxidant resveratrol.

## RESULTS

### Cytotoxic effects of ADC on HUVECs

**Figure 1:**
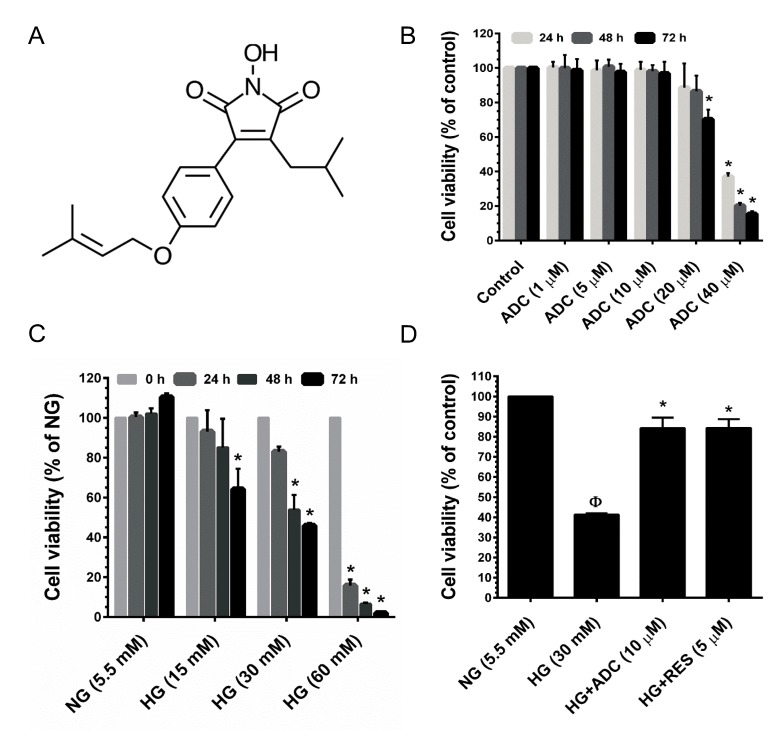
Cytotoxic effect of HG and ADC on HUVECs **A.** Chemical structure of Antrodin C (ADC). **B.** HUVECs were incubated with increasing concentrations of ADC for 24-72 h and the cell viability was measured by MTT assay. The percentage of cell viability was compared with the control (0.01% DMSO) group. **C.** HUVECs were incubated with various doses of HG for 24-72 h and cell viability was measured by MTT assay. The percentage of cell viability was compared with the NG treatment group. **D.** Cells were incubated with HG in the presence or absence of ADC or RES for 72 h. The percentage of cell viability was compared with the NG treatment group. Values represent the mean ± SD of three independent experiments. Statistical significance was set at ^Ф^*P* < 0.05 compared to NG *vs.* HG and *P < 0.05 compared to HG *vs.* samples.

First, the cytotoxicity of ADC was determined. HUVECs were incubated with increasing doses of ADC (1, 5, 10, 20 and 40 µM) for 24, 48 and 72 h, and cell viability was determined by MTT colorimetric assay. Dose-response results showed that ADC does not affect cell viability up to the concentration of 10 µM for 72 h (Figure 1B). Concentrations greater than 10 µM showed a significant reduction in cell viability after 24 h, a similar trend was also observed at 48 and 72 h (Figure 1B). Based on these results, we chose a non-cytotoxic concentration of ADC (10 µM) as the treatment dose for further experiments. HUVECs exposed to HG (15, 30 and 60 mM) for 24-72 h exhibited a dose- and time-dependent reduction in cell viability. Particularly, treatment with 30 and 60 mM for 72 h reduced cell number to 49.3% and 11%, respectively compared to control NG (5.5 mM) cells (Figure 1C). Next, we examined the protective effects of ADC on HG-induced reduction in cell viability. Treatment with ADC (10 µM) significantly reversed the effects of HG in HUVECs. Indeed, compared with HG-only treated cells, the cell viability was markedly increased to 2-fold by co-treatment with ADC for 24-72 h (Figure 1D).

### ADC prevents HG-induced apoptosis in HUVECs

**Figure 2:**
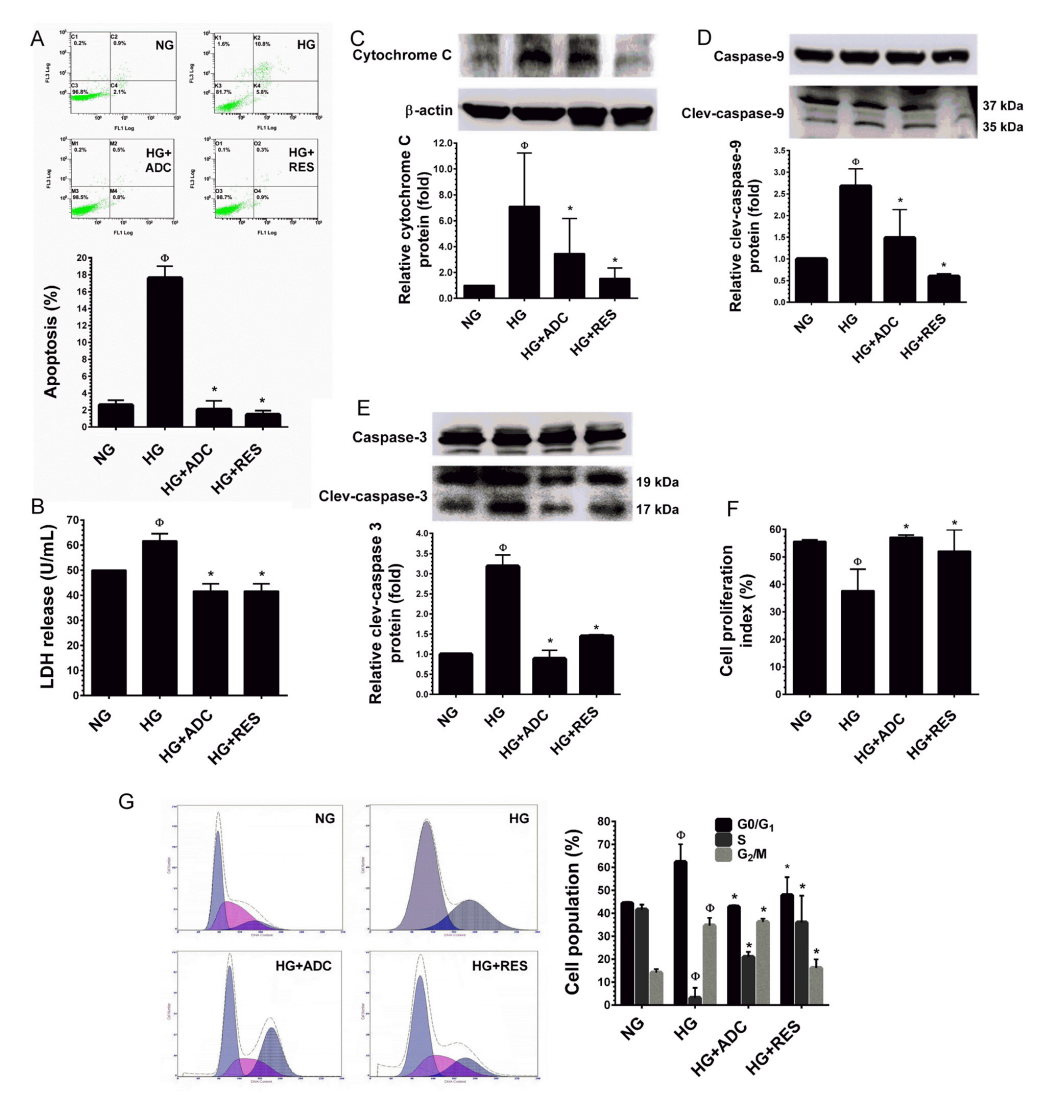
Protective effect of ADC on HG-induced HUVEC apoptosis and growth arrest **A.** HUVECs were incubated with HG in the presence or absence of ADC or RES for 72 h. Apoptotic cell death was performed with Annexin V/PI staining and the hypodiploid DNA was determined by flow cytometry. **B.** HUVECs were treated with HG in the presence or absence of ADC or RES for 72 h. LDH release in the supernatant of HUVEC cultures was measured by LDH assay kit as described in Materials and Methods. **C.**-**E.** Protein expression levels of cytochrome C, caspase-9 and caspase-3 levels were determined by western blot analysis. The relative protein expression of cytochrome C was normalized with β-actin, whereas cleaved capase-9 and cleaved caspase-3 levels were normalized with pro-caspase-9 and pro-caspase-3, respectively. **F.** HUVECs proliferation index was determined by flow cytometry. **G.** HUVECs were incubated with HG in the presence of ADC or RES for 72 h. Cell-cycle distribution was measured by flow cytometer using PI. Percentage of cell population in each transition phase is shown in the histogram. Values represent the mean ± SD of three independent experiments. Statistical significance was set at ^Ф^*P* < 0.05 compared to NG *vs.* HG and *P < 0.05 compared to HG *vs.* samples.

Results of flow cytometric analysis showed that exposure of HUVECs to HG (30 mM) for 72 h resulted in a moderate increase in cell apoptosis, whereas treatment with ADC (10 µM) or RES (5 µM) failed to induce HUVEV apoptosis (Figure 2A). A non-cytotoxic concentration of ADC (10 µM) significantly prevented HG-mediated apoptosis, which was significantly reduced to 2.7% ± 0.7% from 17.6% ± 1% (*P* < 0.05, *vs*. HG alone). In addition, HUVECs incubated with NG (5.5 mM) exhibited a basal level of apoptosis (2.6% ± 0.4%), whereas treatment with ADC (10 µM) or RES (5 µM) even blocked the basal level of apoptosis. The percentage of apoptosis was noted as 1% ± 0.1% and 0.8% ± 0.05%, respectively (Figure 2A). In contrast, exposure of HUVECs to HG for 72 h did not increase LDH level in the culture media suggesting that HG did not cause HUVEC injury or necrosis (Figure 2B). To further clarify the effect of ADC on HG-induced apoptosis, caspase signaling cascade proteins were examined by western blot analysis. As shown in Figure 2C, HG induced loss of mitochondrial membrane potential as evidenced by the increase in cytochrome C protein level (7.09-fold) in the cytoplasm. Apparently, ADC significantly altered the mitochondrial membrane potential as indicated by the decrease in cytochrome C expression to 3.44-fold in HG-induced HUVECs. In addition, HG resulted in significant activation of caspase-9 (2.6-fold) and caspase-3 (3.1-fold) compared to the NG-treated cells, indicating the treatment of HUVECs with HG induced apoptotic cell death. However, caspase-9 and caspase-3 expression levels in HG-induced HUVECs were attenuated to 1.48-fold and 0.88-fold, respectively (Figure 2D, 2E).

### ADC prevents HG-induced growth arrest in HUVECs

Next, to examine whether the reduction in cell number was associated with growth arrest, the effects of ADC on HUVEC proliferation under HG conditions were measured by trypan blue exclusion assay. In line with a previous study [30], HUVECs maintained in the NG condition exhibited significantly greater proliferation as indicated by proliferation index (PI) value of 55.62 ± 0.5% (Figure 2F). However, a dramatic reduction in cell proliferation was observed in the HG condition (37.7 ± 7.7%). The HG-mediated inhibition of cell proliferation was significantly prevented by ADC as shown by the increased PI value of 57.17 ± 0.7% (Figure 2F). Since, reduced cell proliferation is a common hallmark of cell-cycle arrest, we further clarified these results with cell-cycle analysis. Cell-cycle distribution, measured by flow cytometry indicated that treatment with HG arrested HUVECs at the G_1_-S transition phase as evidenced by an increased cell population at the G_0_/G_1_ phase, by 62.3% from 44.4% (NG). This effect was significantly attenuated by co-treatment with ADC which kept the percentage of cells at the G_0_/G_1_ phase near the control value (42.8%) (Figure 2G). To further clarify this effect, cell-cycle regulatory proteins, such as cyclins and cyclin-dependent kinases (CDKs) were examined by immunoblotting. Our results strongly support the notion that G_1_-S transition phase regulatory proteins, particularly cyclin D1, CDK4, CDK6, cyclin E and CDK2 were significantly down-regulated by HG. However, co-treatment with ADC significantly blocked HG-mediated reduction in cyclin and CDK protein levels (Figure 2G). In addition, treatment with HG in the presence or absence of ADC or RES did not show any alteration in protein levels of cyclin B1 and cdc2, which are involved in the G_2_-M transition phase.

### ADC inhibits HG-induced endothelial cell senescence

**Figure 3:**
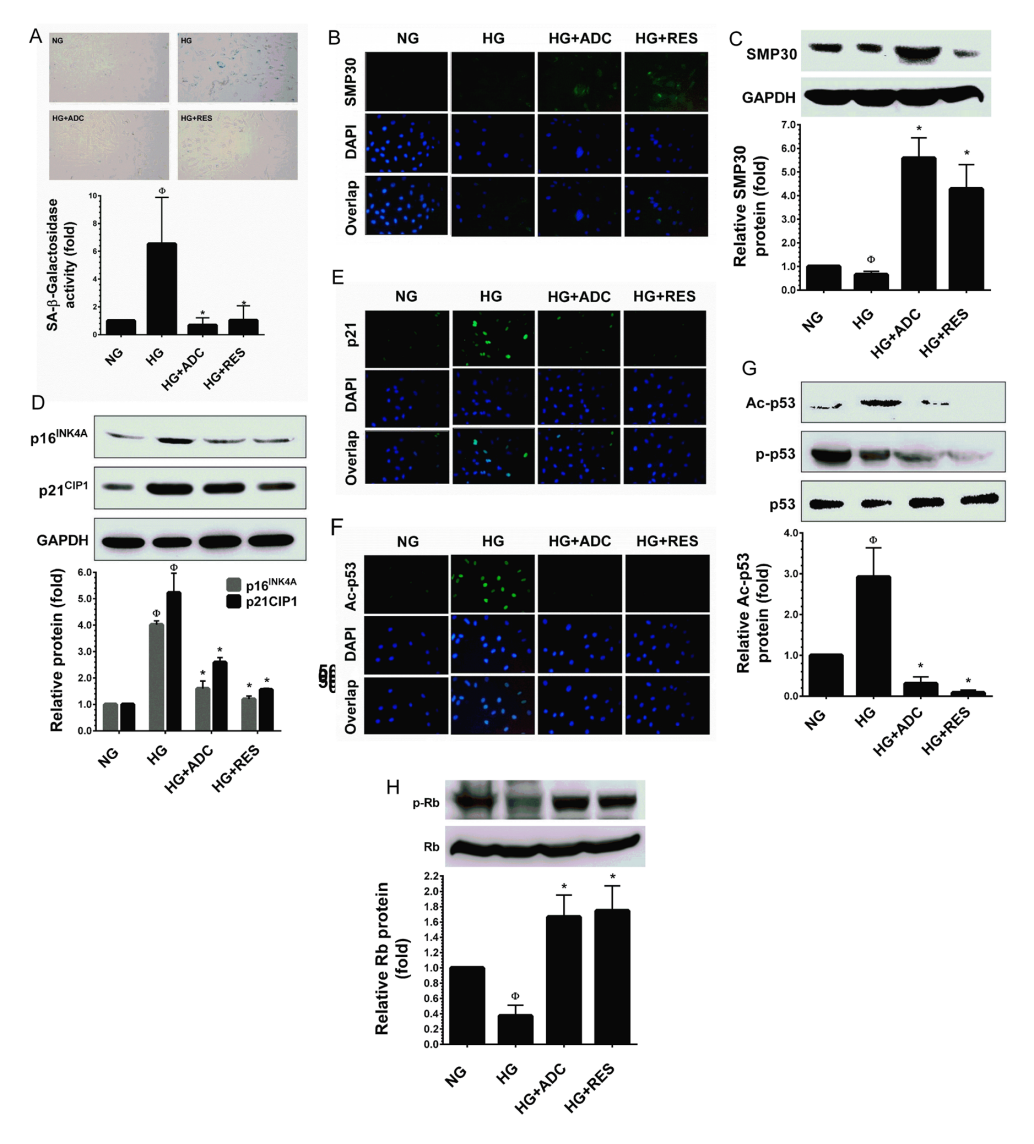
ADC prevents HG-induced senescence in HUVECs To determine the effect of ADC on HG-induced senescence, HUVECs were incubated with HG in the presence or absence of ADC or RES for 72 h. **A.** Cellular senescence was determined by SA-β-gal assay. The top panel shows representative figures and the lower panel shows quantitative analysis of SA-β-gal positive cells per microscopic field. **B.**,**E.**,**F.** The protein expression of SMP30, p21^CIP1^ and acetylated p53 was measured by immunofluroscence using specific primary antibodies and FITC-conjugated secondary antibody (green). The cellular localization of SMP30, p21^CIP1^ and acetylated p53 was photographed using a fluorescence microscope. DAPI was used to stain the nucleus. **C.** SMP30 protein expression level was determined by western blot analysis and the relative SMP30 expression was normalized with GAPDH. **D.** Senescence-associated protein p21^CIP1^ and p16^INK4A^ levels were determined by western blotting and the relative protein levels were normalized with GAPDH. **G.**,**H.** The protein levels of acetylated p53, phosphorylated p53 and phosphorylated Rb levels were measured by western blot analysis. The relative protein levels of ac-p53 and p-Rb were normalized with total p53 and total Rb levels, respectively. Values represent the mean ± SD of three independent experiments. Statistical significance was set at ^Ф^*P* < 0.05 compared to NG *vs.* HG and *P < 0.05 compared to HG *vs.* samples.

A significant increase in SA-β-gal positive HUVECs was observed at 48 h and reached a maximum level at 72 h after treatment with HG. SA-β-gal activity in HUVECs treated with HG reached 6.5 ± 2.3-fold after 72 h, whereas the increased SA-β-gal positive cells were significantly reduced to 0.6 ± 0.3-fold and 1.0 ± 0.7-fold by co-treatment with ADC and RES, respectively (Figure 3A).Senescence marker protein 30 (SMP30), also known as regucalcin, is a 34 kDa cytosolic marker protein for aging. Loss of SMP30 expression was frequently observed in senescent cells [31]. Previous studies have reported that exposure of endothelial cells to HG (30 mM) rapidly decreases SMP30 expression *in vitro* [13, 32]. Therefore, we examined the protein expression level of SMP30 using immunofluorescence analysis. Concomitant with previous studies [13, 32], the endogenous expression of SMP30 was significantly reduced by HG (30 mM) as observed by a reduction in fluorescence positive cells. However, treatment with ADC (10 µM) or RES (5 µM) significantly increased SMP30 expression as evidenced by increased fluorescence positive cells (Figure 3B). This data was further confirmed by western blot analysis. Co-treatment with ADC (10 µM) or RES (5 µM) significantly ameliorated HG-induced reduction in SMP30 protein levels. Interestingly, the increase in SMP30 protein level in ADC or RES treated cells under the HG condition was higher than that at the basal level (Figure 3C).

Increased levels of cyclin-dependent kinase inhibitors (CKIs), such as p16^INK4A^ and p21^CIP1^ were frequently observed in senescent or aging cells [33]. Maeda et al. [34] reported a remarkable increase of p16^INK4A^ and p21^CIP1^ protein levels were noted in endothelial cells exposed to intermediate high glucose (22 mM) for 3 days. Therefore, we further examined whether the effect of ADC on HG-induced HUVEC senescence was associated with parallel changes in the expression of p16^INK4A^ and p21^CIP1^, proteins that are involved in endothelial cell senescence. As we expected, compared with NG-treated cells, the protein expression levels of p16^INK4A^ and p21^CIP1^ were significantly up-regulated by HG (4.02-fold and 5.2-fold, respectively). However, treatment with ADC reduced the protein levels of p16^INK4A^ and p21^CIP1^ to 1.6-fold and 2.5-fold, respectively in HUVECs under HG conditions (Figure 3D). This effect was further confirmed by immunofluorescence analysis. Increased p21^CIP1^ expression levels were observed in HG-only treated cells, whereas low p21^CIP1^ expression levels were noted in ADC or RES treated cells under HG conditions (Figure 3E). During premature cellular senescence, aberrant activation of oncogenes and oxidative stress has been reported. Particularly, when cells are damaged, they withdraw from the cell-cycle and try to repair damage by activating the p53-p21^CIP1^ and retinoblastoma protein (Rb)-p16^INK4A^ pathways [35-37]. To determine whether ADC regulates HUVE senescence *via* altering the p53 pathway, we examined the levels of p53 phosphorylation and acetylation under HG conditions. Results from immunofluorescence analysis revealed that exposure of HUVECs to HG for 72 h significantly increased p53 acetylation as evidenced by increases in fluorescence in HUVECs. However, reduced fluorescence intensity was observed in ADC or RES co-treatment groups, showing that ADC and RES significantly blocked HG-mediated acetylation of p53 (Figure 3F). This effect was further confirmed by immunoblotting. As shown in Figure 3G, compared with the NG treatment group, a remarkable increase in p53 acetylation (Ac-p53) was observed after treatment with HG for 72 h (2.9-fold), whereas increases in Ac-p53 levels were significantly attenuated in the presence of ADC. Indeed, positive control RES-treated cells showed a complete inhibition of Ac-p53 in HUVECs under HG. In contrast to the ADC effect on p53 acetylation, ADC or RES failed to restore HG-induced reduction in p53 phosphorylation (p-p53), indicating that ADC or RES prevent p53 acetylation without affecting its phosphorylation (Figure 3G). Hypo-phosphorylation (active form) of the retinoblastoma protein (Rb) is observed in most senescent cells [38]. To further examine whether ADC modulates high glucose-induced de-phosphorylation of Rb in HUVECs, we monitored the phosphorylated form of Rb (p-Rb) using western blot analysis with specific antibody. A dramatic reduction in Rb phosphorylation was observed after treatment with HG (0.4-fold), whereas co-treatment with ADC significantly prevented HG-mediated de-phosphorylation and increased it to 1.7-fold (Figure 3H). However, the total form of Rb was unaffected by either HG or ADC.

### ADC modulates HG-induced overproduction of ROS in HUVECs

**Figure 4:**
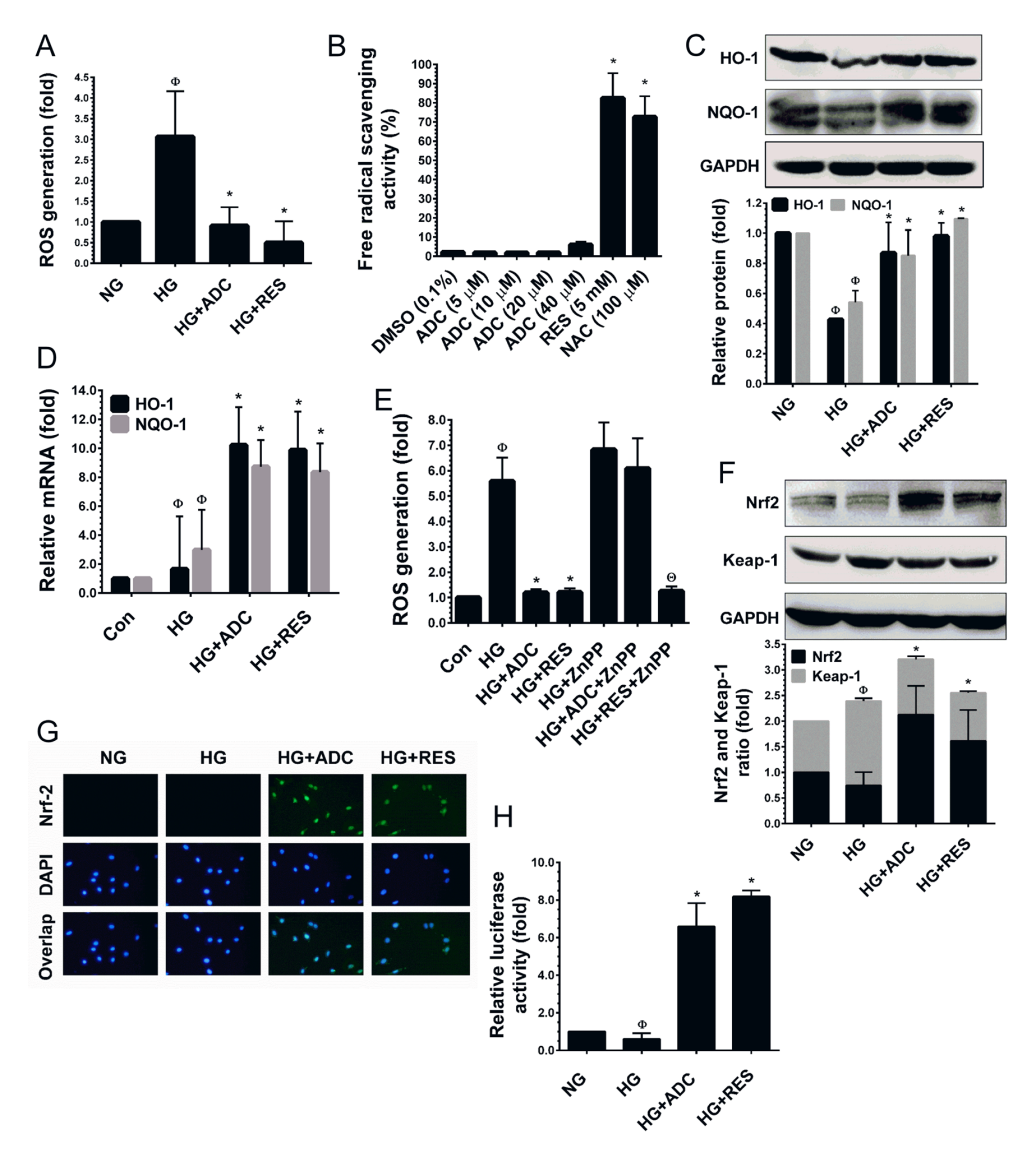
ADC activates Nrf2-dependent anti-oxidant defense in HUVECs **A.** To determine the ROS inhibitory effect of ADC, HUVECs were incubated with HG in the presence or absence of ADC or RES for 24 h. After incubation, intracellular ROS was measured by spectrometer using DCFH_2_-DA flurogenic probe. **B.** The free-radical scavenging activity of ADC was determined by DPPH assay. NAC and RES were used as positive controls. **C.**,**F.** Cells were treated with HG in the presence or absence of ADC or RES for 24 h. The protein levels of HO-1, NQO-1, Nrf2 and Keap-1 were determined by western blot analysis. **D.** To quantify the mRNA expression levels of HO-1 and NQO-1, HUVECs were incubated with HG in the presence or absence of ADC or RES for 24 h. Total RNA was subjected to Q-PCR analysis. Relative mRNA levels were normalized with β-actin mRNA. **E.** HUVECs were pre-treated with ZnPP (10 mM) for 2 h and then incubated with HG in the presence or absence of ADC or RES for 24 h. Intracellular ROS was quantified by spectrophotometer. **G.** The protein expression and nuclear localization of Nrf2 was determined by immunofluorescence analysis. The subcellular and nuclear localization of Nrf2 was photographed using a fluorescence microscope. DAPI was used to stain the nucleus. **H.** To determine the Nrf2 transcriptional activity, HUVECs were transiently transfected with ARE promoter construct using ipofectamine and incubated with HG in the presence or absence of ADC or RES for 6 h. Cell lysates were mixed with luciferase reagents and quantified using an illuminometer. The fold change of relative ARE promoter activity was compared with untreated cells (NG). Values represent the mean ± SD of three independent experiments. Statistical significance was set at ^Ф^*P* < 0.05 compared to NG *vs.* HG, *P < 0.05 compared to HG *vs.* samples and ^Θ^*P* < 0.05 compared to HG *vs.* ZnPP pre-treatment group.

It is well-known that hyperglycemia can increase ROS accumulation, which is the prevailing mechanism leading to endothelial cell dysfunction [13, 39]. Therefore, we hypothesized that ADC could modulate HG-induced senescence in HUVECs through the inhibition of ROS generation. To determine the efficacy of ADC in reducing HG-induced ROS generation, we used a cell-permeable fluorescent dye DCFH_2_-DA which fluorescences upon oxidation by ROS. As shown in Figure 4A, compared with NG-treated cells, a remarkable increase of intracellular ROS (3.06-fold) was observed in HUVECs. However, co-treatment with ADC strongly inhibited this increase reverting ROS to basal levels (0.91-fold) under HG-induced conditions. Indeed, treatment with RES further reduced the basal level of ROS to 0.49-fold lower than that in the HG condition. This data suggests that ADC may possess strong anti-oxidant properties. However, the underlying mechanism of this action was unclear. To unravel the mechanism, next we examined whether ADC possess direct free-radical scavenging ability by performing a cell-free DPPH free radical scavenging assay with various doses of ADC (1-40 µM). As shown in Figure 4B, ADC up to the concentration of 20 µM did not show free-radical scavenging effect in a cell-free system, whereas ADC at 40 µM did show an effect (6.08%) which was statistically not significant to the control group. The known anti-oxidants, NAC and RES exhibited potent free-radical scavenging effects of 82.5% and 64.67%, respectively.

### ADC prevents HG-mediated reduction in HO-1 and NQO-1 expression in HUVECs

Previous studies have demonstrated that *A. cinnamomea* or its components possess strong anti-oxidant effects through acting as direct free-radical scavengers or activators of cellular anti-oxidant genes [23, 25]. Bearing this in mind, we hypothesized that ADC may eliminate intracellular ROS through the induction of anti-oxidant genes. We found that exposure of HUVECs to HG for 72 h reduced the activity of the endogenous anti-oxidant system as evidenced by reduction in protein levels of HO-1 and NQO-1 to 0.42-fold and 0.54-fold, respectively (Figure 4C). However, treatment with ADC significantly prevented the HG-mediated reduction in HO-1 and NQO-1 protein levels and restored them to nearly basal level, 0.86-fold and 0.85-fold, respectively (Figure 4C). This effect was further confirmed by Q-PCR analysis, which showed that treatment with ADC significantly augmented HO-1 and NQO-1 mRNA expression by 10.2-fold and 8.7-fold respectively (Figure 4D). Further, to clarify the importance of ADC-mediated induction of HO-1, ROS inhibition was determined under HO-1 inhibited conditions. HUVECs were pretreated with a pharmacological dose of ZnPP and treated with HG in the presence or absence of ADC or RES. As shown in Figure 4E, an elevated level of ROS (6.8-fold) caused by HG was observed in ZnPP-treated cells. Co-treatment with ADC failed to inhibit the HG-induced ROS generation in ZnPP-treated cells (6.09-fold). This data clearly demonstrates that HO-1 plays a central role in ADC-mediated anti-oxidant effects.

### ADC increases Nrf2 transcriptional activity in HG-induced HUVECs

It has been well demonstrated that HO-1 and NQO-1 are activated by Nrf2, a major transcription factor regulating ARE-dependent anti-oxidant gene expression [22]. We, therefore, attempted to determine whether ADC-mediated HO-1 and NQO-1 expression are associated with Nrf2 activity. As shown in Figure 4F, HUVECs exposed to HG reduced the levels of Nrf2 protein, whereas ADC or RES increased the protein level to 2.1-fold and 1.6-fold above the basal level, respectively. In addition, we found that HG exposure significantly increased the protein level of Keap-1 (1.64-fold), an endogenous inhibitor of Nrf2. However, treatment with ADC significantly inhibited Keap-1 expression (1.07-fold), this is similar to the basal level (Figure 4F). Next, to determine the transcriptional activity of Nrf2, we monitored the nuclear translocation of Nrf2 by immunofluorescence analysis. Immunofluorescence staining showed that there was no detectable Nrf2 signal in HG-treated cells in either the cytoplasm or nucleus. A basal level of Nrf2 in the cytoplasm was observed in the control cells (NG). Indeed, nuclear accumulation of Nrf2 was significantly increased when the cells were co-treated with ADC (Figure 4G). Furthermore, to demonstrate that ADC facilitates Nrf2 transcriptional activity in HUVECs under the HG condition, we used the ARE-dependent luciferase reporter system. As shown in Figure 4H, the luciferase activity in HUVECs transfected with the ARE reporter construct was significantly reduced by HG (0.6-fold), whereas a 6.5-fold increase in luciferase activity was observed in the ADC co-treatment group. This data strongly supports our hypothesis that ADC eliminates HG-induced ROS generation through the activation of Nrf2-dependent anti-oxidant genes.

### ADC failed to modulate HG-mediated dysfunction in Nrf2 silenced HUVECs

**Figure 5:**
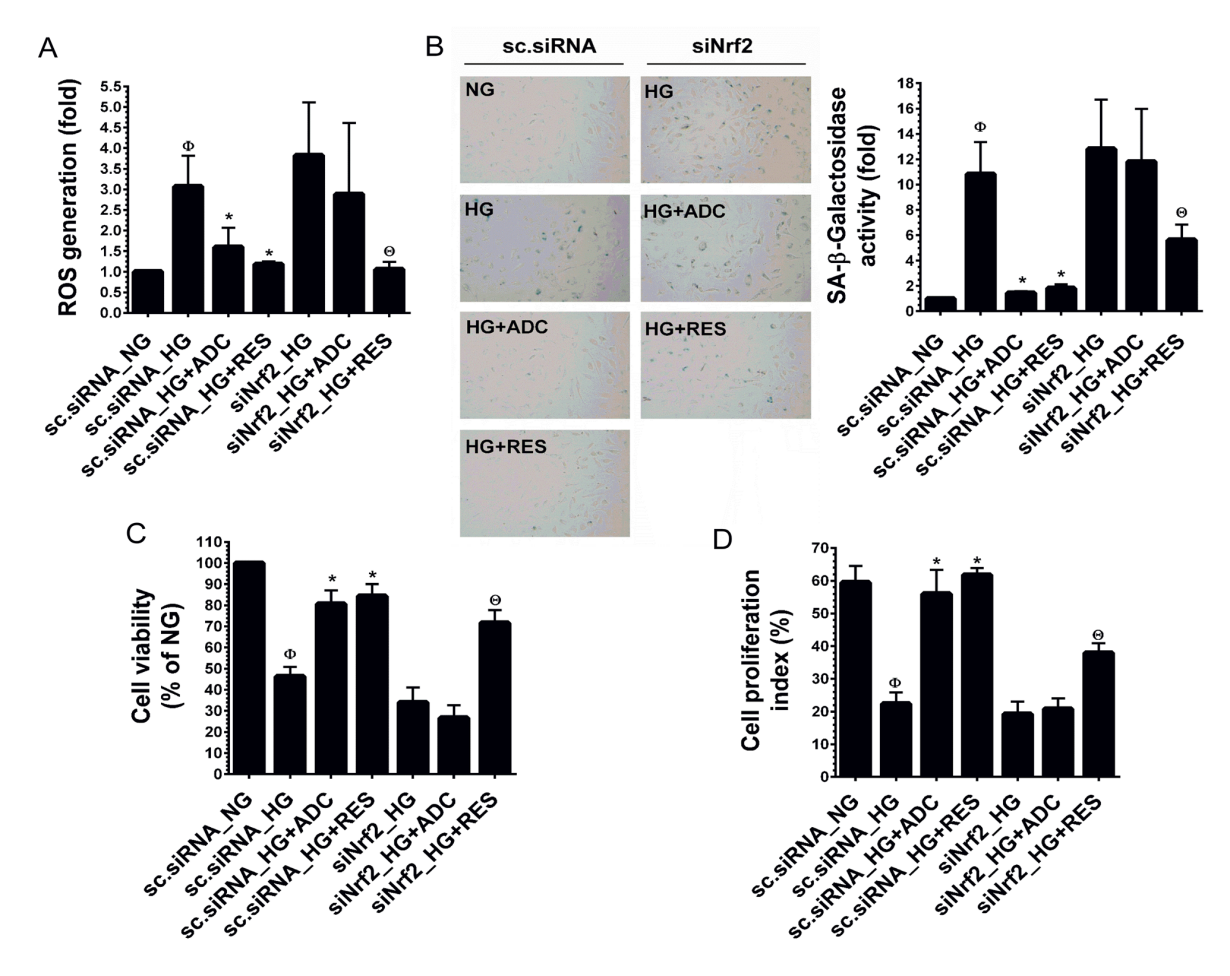
ADC has a protective effect in Nrf2 silenced cells HUVECs were transfected with specific siRNA against Nrf2 or scrambled siRNA. After transfection for 24 h, cells were incubated with HG in the presence or absence of ADC or RES for 24-72 h. **A.** Intracellular ROS was measured by DCFH_2_-DA assay using a fluorescence spectrophotometer. Total RNA was extracted and subjected to Q-PCR analysis to determine HO-1 and NQO-1 mRNA expression levels. **B.** SA-β-gal activity was determined by SA-β-gal assay kit after incubation for 72 h. The left panel shows representative figures and right panel shows quantitative analysis of SA-β-gal positive cells per microscopic field. **C.** Cell viability was determined by MTT colorimetric assay. **D.** Cell proliferation index was determined by flow cytometry. Values represent the mean ± SD of three independent experiments. Statistical significance was set at ^Ф^*P* < 0.05 compared to NG *vs.* HG, *P < 0.05 compared to HG *vs.* samples and ^Θ^*P* < 0.05 compared to HG *vs.* siNrf2 transfection group.

To demonstrate the involvement of Nrf2 in ADC-mediated anti-oxidant effects, we developed an Nrf2 gene knockdown model using Nrf2 siRNA. As shown in Figure 5A, a 3-fold increase in ROS generation in response to HG was observed in scrambled siRNA (control siRNA) transfected cells, whereas co-treatment with ADC significantly reduced this increase to 1.5-fold. Although, treatment with HG in the presence or absence of ADC showed a 3.8-fold increase of ROS generation in siNrf2 transfection. This increase was slightly reduced by ADC (2.8-fold) under similar conditions. Interestingly, treatment with RES significantly reduced ROS generation in both control siRNA or siNrf2-transfected cells, which may be attributed to its direct ROS scavenging ability. To further clarify this protective effect, HG-induced senescence was measured by SA-β-gal staining. In control siRNA transfected cells, a 10-fold increase of SA-β-gal activity was observed compared with NG-treated cells, whereas co-treatment with ADC significantly blocked this effect and reduced SA-β-gal activity to 1.4-fold (Figure 5B). However, a remarkable increase in SA-β-gal activity (12.8-fold) was observed in siNrf2 knockdown cells under HG. Co-treatment with ADC failed to inhibit HG-induced SA-β-gal activity in siNrf2 knockdown cells as evidenced by 11.8-fold SA-β-gal activity (Figure 5B). Next, to examine the involvement of ADC-mediated Nrf2 activation in HG-induced growth arrest, proliferation and cell-cycle analysis were performed. As shown in Figure 5C, compared to HG-treated cells (46.3%), co-treatment with ADC significantly increased the number of viable cells to 80.6% in control siRNA transfected cells, whereas the percentage of viable cells in siNrf2 transfected cells declined to 34% by HG. ADC failed to recover the HG-mediated reduction in cell number as shown by only 26.6% of siNrf2 transfected cells being viable. A similar trend was also observed in cell proliferation index: the percentage of cell proliferation in HG-treated scsiRNA and siNrf2 transfected cells declined to 22.3 ± 3.4% and 19.2 ± 3.8%, respectively, whereas cells were transfected with scsiRNA under NG condition showed 59.5 ± 5.0% of proliferation. ADC treatment also did not exhibit any significant increase in cell proliferation under the siNrf2 system (20.8 ± 3.12%) whereas a significant increase in cell proliferation was observed in ADC-treated control siRNA transfected cells (56.03 ± 7.2% (Figure 5D). These data strongly suggest that ADC-mediated induction of anti-oxidant genes modulates HG-induced endothelial cell dysfunction.

### ADC protects HUVECs from hyperosmolality-induced apoptosis

**Figure 6:**
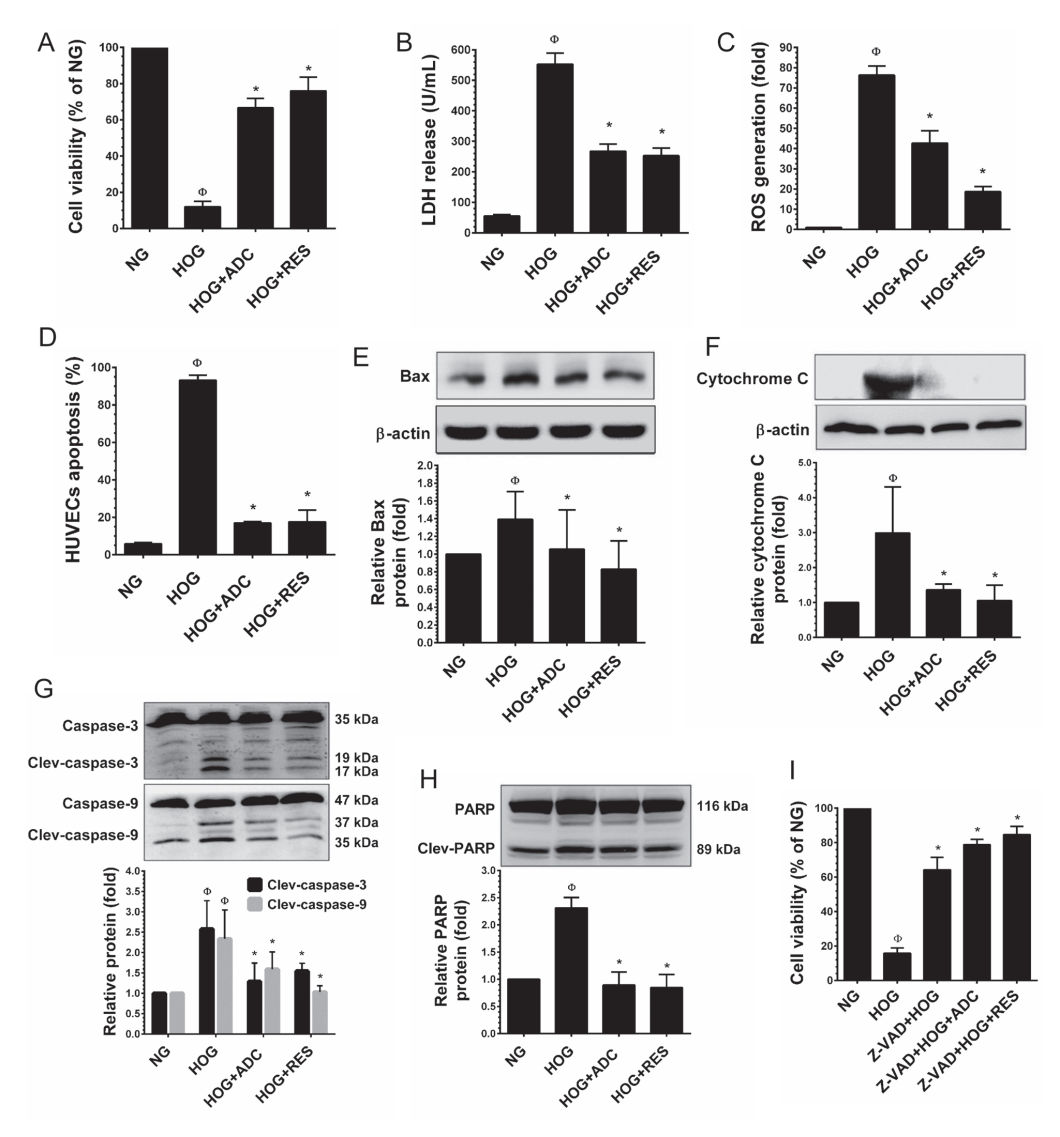
ADC protects HUVECs from hyperosmotic glucose (HOG)-induced cell death HUVECs were incubated with HOG in the presence or absence of ADC or RES for 24 h. **A.** Cell viability was determined by MTT colorimetric assay. **B.** HUVEC injury was determined by LDH release into the culture media. **C.** Intracellular ROS generation was quantified by DCFH_2_-DA flurogenic assay. **D.** Apoptosis was determined by Annexin V/PI staining assay. **E.**,**F.** Protein levels of Bax and cytochrome C levels were measured by western blot analysis. The relative protein levels of Bax and cytochrome C were normalized with β-actin. **G.**,**H.** The activation (cleaved form) of caspase-9, caspase-3 and PARP were determined by western blot analysis and the protein levels were normalized with their corresponding pro-form. **I.** HUVECs were pre-incubated with caspase-3 inhibitor (Z-VAD-FMK, 30 µM) for 2 h and then incubated with HG in the presence or absence of ADC or RES for 24 h. The cell viability was determined by MTT assay. Values represent the mean ± SD of three independent experiments. Statistical significance was set at ^Ф^*P* < 0.05 compared to NG *vs.* HG and *P < 0.05 compared to HG *vs.* samples.

It has been demonstrated that exposure of HUVECs to a hyperosmolar concentration of glucose (HOG, 60 mM) for 24-48 h, increased rapid apoptosis and HUVEC injury. Therefore, we further examined whether ADC could protect endothelial cells from hyper glucose-induced cell death. As shown in Figure 6A, exposure of HUVECs to HOG for 24 significantly reduced the cell viability to 12%, which was significantly protected by ADC and the cell viability was increased to 66.6%. In addition, an elevated level of LDH (552.3 units/mL) in culture media was observed when HUVECs were incubated with HOG for 24 h. However, co-treatment with ADC significantly prevented HOG-induced HUVEC injury as evidenced by decreased level of LDH (267.3 units/mL) in the culture media (Figure 6B). Further analysis revealed that HOG-mediated cell death was strongly associated with over production of ROS as evidenced by the 76.3-fold increase ROS generation observed. Whereas, co-treatment with ADC significantly ameliorated HOG-induced ROS generation to 42.6% (Figure 6C). Next to examine the anti-apoptotic effect of ADC, HUVECs were incubated with HOG in the presence or absence of ADC or RES for 24 h, apoptotic cell death and LDH release were measured. As shown in Figure 6D, exposure of HUVECs to HOG for 24 h resulted in a significant increase in cell apoptosis (74%), whereas co-treatment with ADC prevented HOG-induced HUVEC apoptosis, which was significantly reduced to 31%. Since, the over production of intracellular ROS and mitochondrial damage are inevitable, we examined the activation of the intrinsic apoptosis regulatory proteins. An elevated level of Bax, a mitochondrial membrane-associated apoptotic protein was found in HOG-treated cells (2.3-fold), which was significantly inhibited by ADC. Indeed, ADC reduced the Bax expression to below basal levels (0.89-fold) under the HOG condition (Figure 6E). In addition, compared with control, a remarkable increase of cytochrome C (3.9-fold) was observed in HOG treated cells, however co-treatment with ADC significantly reduced to 1.47-fold (Figure 6F). We further examined the expression level of cytochrome C downstream effector caspases such as caspase-9 and caspase-3. Results showed that a 2.3-fold and 2.5-fold increase in active (cleaved) caspase-9 and caspase-3, respectively, were observed in HOG-treated cells, whereas co-treatment with ADC reduced them to 1.5-fold and 1.29-fold, respectively (Figure 6G). Moreover, following the activation of caspase-3, an increase in PARP activation (2.3-fold) was observed in HOG treated cells that was significantly attenuated by ADC (0.89-fold) (Figure 6H). To further confirm that the HOG-induced cell death was mediated by activation of caspase signaling events, HUVECs were pre-incubated with Z-VAD-FMK (30 µM), a pharmacological inhibitor of caspase-3 for 2 h and treated with HOG in the presence or absence of ADC or RES for 24 h. As shown in Figure 6I, compared with HOG-treated cells (15.8%), the percentage of viable cells were significantly increased to 64.2% in Z-VAD-FMK-treated cells, which showed that HOG-induced cell death was mediated by the caspase cascade. Interestingly, the percentage of viable cells was further increased to 78.8% and 84.6%, when co-incubated with ADC and RES, respectively.

### Nrf2-mediated anti-oxidant genes required for anti-apoptotic effect of ADC

**Figure 7:**
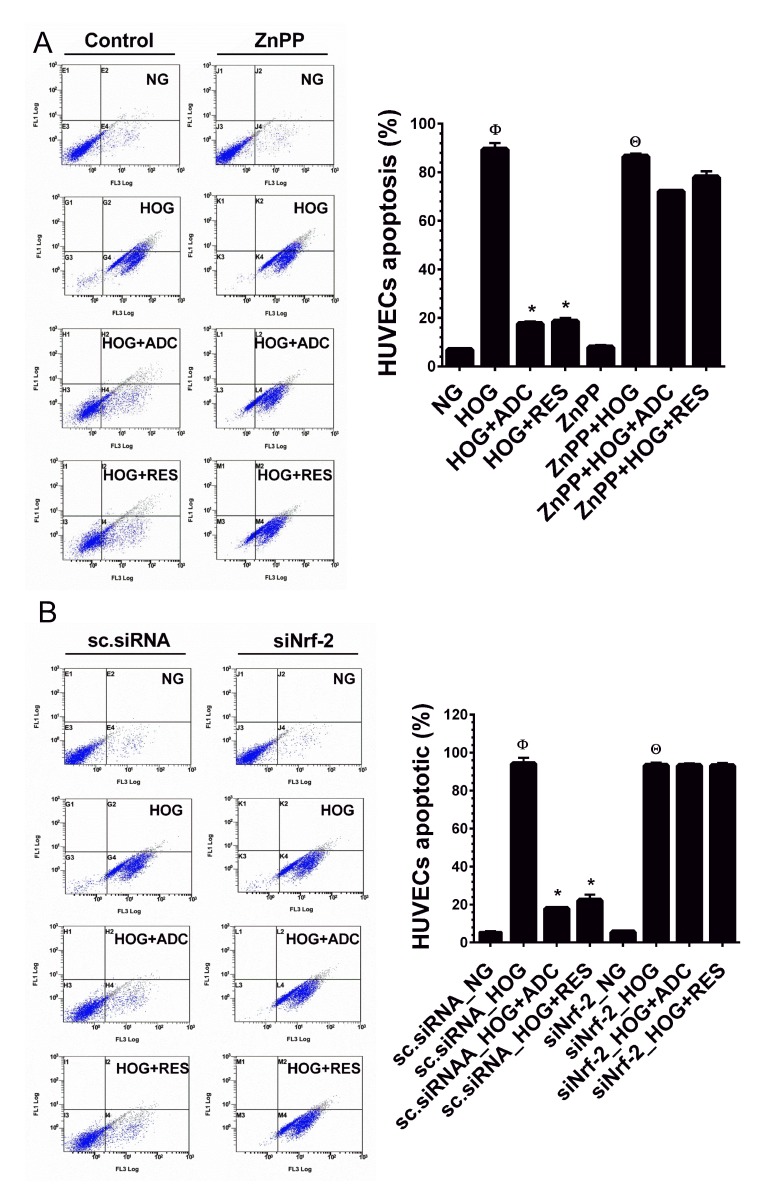
ADC failed to protect HOG-induced HUVEC apoptosis under Nrf2 silenced conditions **A.** HUVECs were transfected with specific siRNA against Nrf2 or scrambled siRNA (control). After transfection for 24 h, cells were incubated with HOG in the presence or absence of ADC or RES for 24 h. HUVEC apoptosis was determined by Annexin V/PI staining using a flow cytometer. **B.** HUVECs were pre-treated with ZnPP for 2 h and then incubated with HOG in the presence or absence of ADC or RES for 24 h. Apoptotic cell death was quantified by flow cytometry. Values represent the mean ± SD of three independent experiments. Statistical significance was set at ^Ф^*P* < 0.05 compared to NG *vs.* HG, *P < 0.05 compared to HG *vs.* samples and ^Θ^*P* < 0.05 compared to NG *vs.* HOG in siNrf2 transfected cells.

To further explain our hypothesis that Nrf2-mediated anti-oxidant genes, at least HO-1 is required for ADC-mediated anti-apoptotic effects. HUVECs were pre-treated with ZnPP for 2 h and then incubated with HOG for 24 h in the presence or absence of ADC or RES. The cell apoptosis was measured by flow cytometry. As shown in Figure 7A, compared to control cells (6.8%), the percentage of apoptotic cells were markedly increased to 89.4% in HOG-treated cells and reduced to 17.5% by ADC. Whereas, ADC failed to protect HOG-induced apoptosis in ZnPP-treated cells. A similar trend was also observed in siNrf2 knockdown cells in which ADC barely prevented HOG-induced apoptosis (Figure 7B). Essentially, these results strongly suggest that ADC-mediated induction of anti-oxidant genes play a central role on its protective effects.

## DISCUSSION

Cardiovascular disease (CVD) is one of the leading causes of death worldwide, and diabetes mellitus has been implicated as a major risk factor in the development of CVD [40]. Given that hyperglycemia-induced endothelial cell dysfunction is a key event in the onset and progression of CVD, protection of vascular endothelial cells from such dysfunction represents an important strategy for the diagnosis of CVD. The medicinal fungus *A. cinnamomea* and its bioactive components exhibit a variety of bioactivities, including anti-diabetic and anti-hyperlipidemic properties *in vivo* [41]. We previously reported that Antrodin C, a maleimide derivative isolated from the mycelia of *A. cinnamomea* inhibits TGF-β-induced breast cancer cell metastasis *via* inhibition of epithelial-to-mesenchymal transition *in vitro* [29]. Another study shows that ADC inhibits hepatitis C virus (HCV) protease activity *in vitro* [42]. However, other biological effects of this potentially beneficial compound are not well elucidated. In the present study, we used an *in vitro* model of HUVECs to investigate the protective effects of ADC on high glucose-accelerated endothelial cell dysfunction. To our knowledge this is the first report demonstrating that ADC can inhibit hyperglycemia-induced endothelial cell dysfunction *via* inhibition of ROS generation, senescence, growth arrest and apoptosis in cultured HUVECs. These data indicate that ADC might be a potential candidate for the prevention of diabetic-associated vascular complications.

In line with previous studies [30, 43, 44], HUVECs exposed to HG exhibited a dose- and time-dependent reduction in cell viability. The anticipated HG-induced reduction in HUVEC viability was significantly restored by ADC. A similar effect was also observed with RES, the known anti-oxidant treatment group. In addition, we found that HG inhibited the proliferation of HUVECs, and this effect was significantly blocked by ADC. It has been well demonstrated that HG triggers endothelial cell apoptosis in a selective and specific manner, since endothelial cell apoptosis was barely observed in cells treated with the osmotic glucose mannitol [13, 44]. Furthermore, previous reports indicate that HG (30 mM), which is an ambient dose for inducing endothelial cell apoptosis, failed to cause apoptosis in other standardized cells lines such as K-562 (leukemia), P815 (mast cell) YT (nature killer cell) and CCD966SK (skin fibroblast) [45, 46]. In this study, we found that incubation of HUVECs with HG (30 mM) for 72 h significantly increased apoptosis (17.6%), whereas a remarkable and rapid increase of apoptosis (74%) was observed when HUVECs were incubated with very high dose of glucose (60 mM). This increase of apoptosis was achieved within 24 h. Furthermore, HG-mediated endothelial cell necrosis, another form of cell death was measured by LDH release assay. We found that HG (30 mM), which induced apoptosis failed to cause necrosis of HUVECs as evidenced by unaltered levels of LDH. However, incubation of HUVECs with very high concentration of glucose (60 mM) for 24 h resulted in a hyperosmolarity-associated HUVEC necrosis, indicated by elevated levels of LDH. These results were highly consistent with a previous report in which HG (30 mM) induced apoptosis in HUVECS without causing HUVEC necrosis, whereas treatment with HG (60 mM) remarkably increased HUVEC apoptosis as well as necrosis, which was not protected by taurine, an organic compound widely found in animal tissues [44]. Interestingly, co-treatment with ADC prevents both HG (30 mM) and HG (60 mM)-induced HUVEC apoptosis and necrosis without any compromise.

It is well-known that mitochondria play a pivotal role in cell death transduction because loss of membrane integrity (∆Ψm) causes the release of cytochrome C into the cytosol, which subsequently triggers caspase cascade activation [47]. Therefore, detection of the mitochondrial membrane potential provides an early detection of the initiation on apoptosis. Earlier studies revealed that exposure of endothelial cells to HG increase loss of membrane potential and eventually cause apoptosis [39]. According to the data, we found that HUVECs exposed to HG increase mitochondrial membrane permeability as evidenced by increase in cytochrome C and Bax protein levels in the cytosol. However, HUVECs treated with ADC significantly reduced mitochondrial membrane potential as indicated by reduction in cytochrome C and Bax levels. Activation of caspase-3 resulted in DNA fragmentation, a characteristic feature of cell apoptosis [47]. Ho et al. [48] reported that in HUVECs incubated with HG, a significant increase in cleavage of initiator caspases, such as caspase-9 and effector caspases like caspase-3 were observed. In addition, Garcia Soriano et al. [49] demonstrated that endothelial cells incubated in HG exhibited an over activation of PARP, a nuclear enzyme response to DNA injury. In the present study, HG-induced increases in caspase-3, caspase-9 and PARP activation were significantly prevented by ADC. This probably explains the anti-apoptotic functionality of ADC on HUVECs induced by hyperglycemia. An earlier study demonstrated that compared with pathological concentration of high glucose (30 mM), a hyperosmolarity concentration of glucose (HOG, 60 mM) rapidly induced endothelial cell injury or necrosis [44]. The most important finding in this study is that ADC significantly prevents HUVECs from HOG-induced over production of ROS and cellular injury or necrosis as evidenced by reduced levels of LDH because, other anti-oxidants such as taurine failed to rescue hyperosmolarity-induced endothelial cell injury and necrosis [44].

Senescence of vascular endothelial cells plays a critical role in the pathogenesis of cardiovascular diseases, and is mainly caused by ageing, diabetes and stress [50]. Several markers are available to identify senescent cells, of which the most widely used biomarker for senescent and aging cells is senescence-associated β-galactosidase (SA-β-gal) activity, which is defined as SA-β-gal activity which is detectable at pH 6.0 in senescent cells [51]. In line with previous studies [13, 46], the present study also showed more SA-β-gal positive cells in the HG treatment group than in the NG treated group, which was significantly reversed by co-administration of ADC. Since, growth arrest is a common hallmark of cellular senescence, we examined cell-cycle progression using flow cytometry. In agreement with previous observations [52, 53], our study indicates that incubation of HUVECs with HG for 72 h determined a consistent reduction in cell proliferation by arresting cells at the G_1_-S transition phase, as evidenced by increasing the percentage of cells at the G_0_/G_1_ phase. However, at the same time, co-treatment with ADC significantly improved HUVEC proliferation and inhibited HG-mediated growth arrest. However, the underlying mechanisms by which HG-induced apoptosis and growth arrest in HUVECs need to be elucidated. Hyperglycemia-induced ROS production has been implicated as one of the mediators that triggers apoptosis and growth arrest in various cell types including endothelial cells [39, 44, 46]. Although, impaired anti-oxidant defense increased oxidative stress and contributed to the development of diabetic CVD. Thus, scavenging of intracellular ROS by membrane permeable anti-oxidants or activating endogenous anti-oxidative defense by external stimuli could provoke HG-induced oxidative stress, thereby blocking endothelial cell dysfunction [19].

Mounting evidence indicates that natural products are the major source of anti-oxidants, such as flavonoids, terpenoids, quinones, caratinoids, vitamins, polyphenols and polysaccharides. These components act as free-radical scavengers and also regulate anti-oxidative pathways and gene expression patterns. In addition, recent reports also indicate that ergothionene, a type of amino acid protects endothelial cells from HG-induced oxidative stress and cellular senescence through the direct inhibition of intracellular ROS [54, 55]. Therefore, we further investigated whether ADC acts as a direct antioxidant that scavenges or quenches free radicals or eliminates intracellular ROS by activation of the endogenous anti-oxidative defense system. Because, several lines of evidence indicated that components of *A. cinnamomea* exhibited potent anti-oxidative effects by direct free-radical scavenging or induction of anti-oxidant genes [23-25, 46, 56], initially, a cell-free DPPH assay was performed to examine direct free-radical scavenging activity of ADC. The results of DPPH assay showed that ADC does not scavenge free-radicals up to a dose of 20 µM, a statistically not significant increase of free-radical scavenging activity was observed at 40 µM, which is correlated with our previous work in which we reported that antcin M, a steroid like compound isolated from *A. cinnamomea* failed to scavenge free-radicals in a cell-free system, whereas inhibits ROS generation through the activation of Nrf2-dependent antioxidant genes [46]. RES and NAC are known anti-oxidants that exhibit significant free-radical scavenging activity in cell-free systems [57, 58]. These results prompted us to further examine the effects of ADC on the endogenous anti-oxidative defense mechanism, particularly, HO-1, a microsomal enzyme that catalyzes oxidation of heme into anti-oxidant molecules, biliverdin and carbon monoxide. In other hand, NQO-1, a detoxification agent remove quinonoid-medicated ROS generation in the biological systems [22]. Thus, HO-1 and NQO-1 has been recognized as an important therapeutic target of oxidative stress diseases. Growing evidence indicates that acute increase in glucose is accompanied by reduction in endogenous anti-oxidant genes that may result in endothelial cell dysfunction [59]. Our results also show a dramatic reduction in HO-1, NQO-1 and Nrf2 levels upon HG treatment. However, as we expected, ADC up-regulated HO-1 and NQO-1 expression in HG-treated HUVECs, thus decreasing ROS generation. Nrf2, a b-ZIP transcription factor regulates the expression of anti-oxidant genes, including HO-1 and NQO-1 [22]. We found a reduction in Nrf2 expression under HG conditions, whereas ADC up-regulated Nrf2 expression and promoted its nuclear translocation and transcriptional activity. In addition, ADC-mediated HO-1 and NQO-1 expression were significantly blocked by siNrf2, indicating that ADC prevented HG-induced endothelial cell dysfunction through the Nrf2-mediated endogenous anti-oxidant system. However, this observation contradicted a previous report that increases of HO-1, NQO-1 and Nrf2 expression levels were observed in mouse mesangial cells which were exposed to HG for 48 h [60]. These findings suggest that Nrf2-mediated anti-oxidant gene expression in the HG condition varies according to cell type as well as treatment condition.

**Figure 8:**
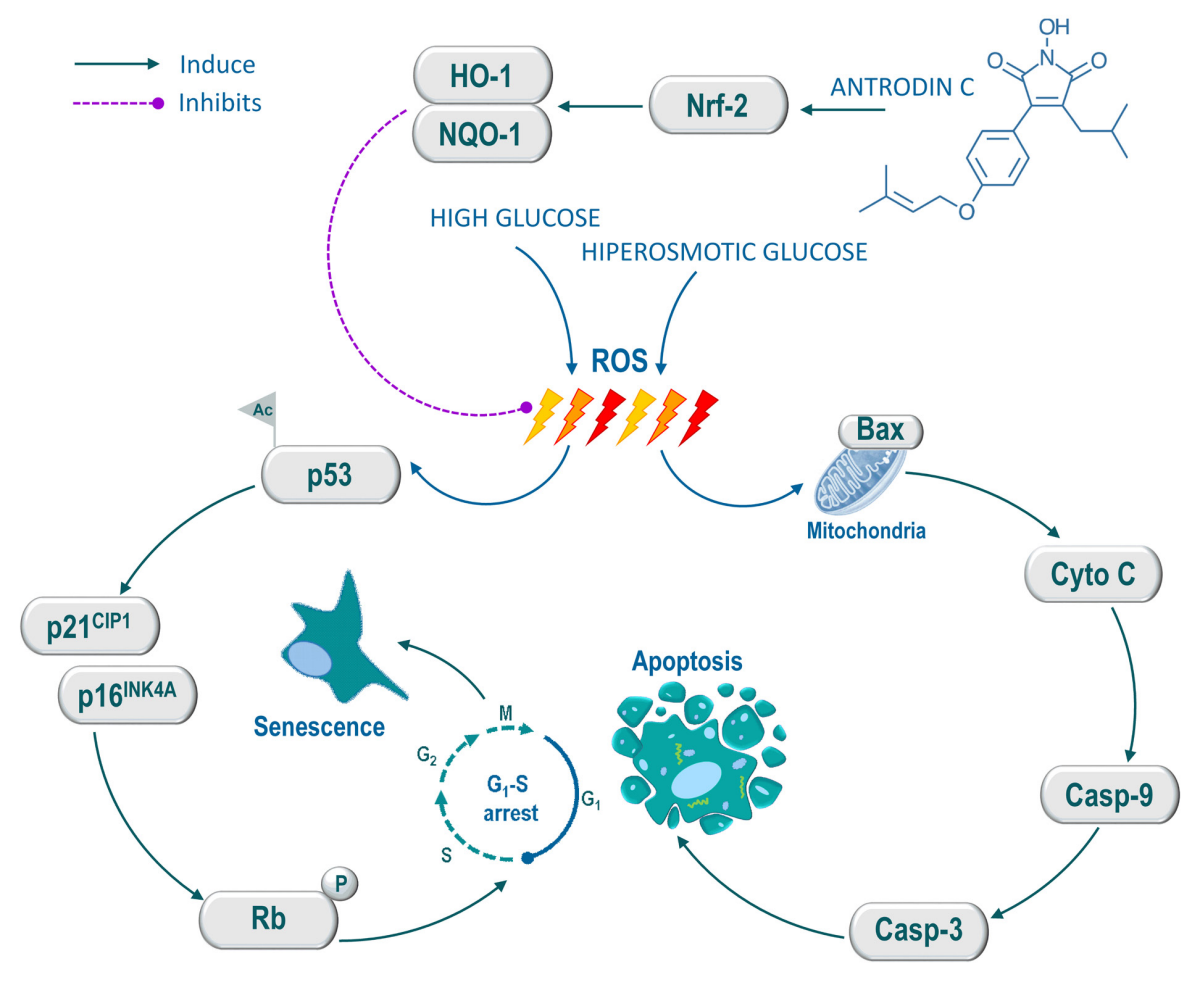
Schematic representation of antrodin C-mediated protection against high glucose or hyperosmotic glucose-induced senescence and apoptosis in human endothelial cells High glucose induces intracellular ROS, which triggers p53 acetylation. Activated p53 up-regulates p16^INK4A^ and p21^CIP1^, which further activates Rb, thereby the cell-cycle was arrested at G_1_-S transition and induced cellular senescence. Conversely, hyperosmotic glucose induce aberrant cellular ROS production, which eventually induce apoptosis in HUVECs. However, treatment with antrodin C activates Nrf2-dependent anti-oxidant genes such as HO-1 and NQO-1 followed by degradation of Keap-1, which facilitates ROS inhibition and its down-stream cascades including cell-cycle arrest, senescence and apoptosis in human endothelial cells.

In conclusion, the present study provides experimental evidence that ADC prevents pathologically high glucose-induced endothelial cell dysfunction *via* blocking intracellular ROS generation, senescence, growth arrest and apoptosis. Our mechanistic evidence suggest that the prevention of endothelial cells likely to result from up-regulation of Nrf2-dependent anti-oxidant genes, particularly HO-1 and NQO-1, which eventually eliminates high glucose-induced oxidative stress and apoptosis of endothelial cells (Figure 8). Our study suggests that Nrf2 activation by ADC may represent a promising intervention in pathologically high glucose-accelerated endothelial dysfunction as well as vascular complications.

## MATERIALS AND METHODS

### Chemicals and reagents

Antrodin C was isolated from the mycelia of *A. cinnamomea* as described previously [26]. The purity of ADC was above 99% according to HPLC and ^1^H-NMR analyses. M199 medium, fetal bovine serum (FBS), sodium pyruvate, penicillin and streptomycin were obtained from Invitrogen (Carlsbad, CA). Endothelial cell growth supplement (ECGS), heparin sodium salt, d-Glucose, 2’, 7’-dichlorofluorescein diacetate (DCFH_2_-DA), 3-(4,5-dimethyl-thiazol-2-yl)-2,5-diphenyl tetrazolium bromide (MTT), zinc protoporphyrin (ZnPP) and resveratrol (RES) were purchased from Sigma-Aldrich (St. Louis, CA). Antibodies against phos-Rb, cyclin D1, cyclin E, CDK2, CDK4, CDK6, Acetyl-p53, p16^INK4A^, p21^CIP1^, phos-p53, Keap-1, Pro-caspase-3, Clev-caspase-3, Pro-caspase-9, Clev-caspase-9, Pro-PARP, Clev-PARP, Cytochrome C and Bax were obtained from Cell Signaling Technology, Danvers, MA. Antibodies against HO-1, NQO-1 and Nrf2 were purchased from Abcam, Cambridge, UK. Antibodies against SMP30, p53 and β-actin were obtained from Santa-Cruz Biotechnology, Dallas, TX. All other chemicals were reagent grade or HPLC grade and supplied by either Merck (Darmstadt, Germany) or Sigma-Aldrich.

### Cell culture and sample treatment

Human umbilical vein endothelial cells (HUVECs) were obtained from the Bioresource Collection and Research Center (BCRC), Hsinchu, Taiwan. HUVECs were grown in M199 medium supplemented with 10% FBS, 30 µg/mL ECGS, 25 U/mL heparin, 2 mM _L_-glutamine, 1.5 g/L sodium bicarbonate and 100 U/L penicillin and streptomycin at 37°C in a humidified atmosphere of 5% CO_2_. Hyperglycemia treatment was induced by treating HUVECs with 15, 30 and 60 mM of d-glucose for 24-72 h. HUVEs were also incubated with HG in the presence or absence of ADC (10 µM) or resveratrol (5 µM). Controls were performed in the presence of media with normal glucose (NG, 5.5 mM).

### Cell viability assay

HUVECs (5 × 10^4^ cells/well) were seeded in a 24-well culture plate and incubated with NG (5.5 mM) or HG (15, 30 and 60 mM) for 24-72 h in the presence or absence of ADC (1-20 µM) or RES (5 µM). Cell viability was measured by MTT colorimetric assay. Briefly, after treatment, the culture media was withdrawn and cells were incubated with MTT (1 mg/mL) in fresh medium for 2 h at 37°C. The MTT formazan crystals were dissolved in 400 µL of dimethyl sulfoxide (DMSO) for each well. The optical density (OD) value was measured at an absorption wavelength of 570 nm (A_570_) using an ELISA microplate reader (Bio-Tek Instruments, Winooski, VT). The percentage of cell viability (%) was calculated as (A_570_ of treated cells/A_570_ of untreated cells) × 100. Independent experiments were repeated three times and the cell viability was normalized to the NG control.

### Quantification of apoptosis

Apoptotic cell death was measured according to the percentage of cells with hypodiploid DNA by using Annexin V and PI binding staining with an Annexin V/PI Apoptosis Detection Kit (BD Bioscences, San Jose, CA). HUVECs (5 × 10^5^ cells/dish) were seeded in a 10 cm culture dish and treated with HG (30 or 60 mM) in the presence or absence of ADC (10 µM) or RES (5 µM) for 72 h. The control group received NG (5.5 mM) alone over the same time course. After treatment, cells were washed twice with PBS and collected using 0.25% trypsin without EDTA, cells were pooled by centrifuging at 1500 × *g* for 5 min. Cells were then re-suspended in 500 μL of binding buffer which contained 1 μL Annexin V-FITC and 5 μL PI and incubated for 5 min in the dark. The stained cells were analyzed directly using a flow cytometer (Beckman Coulter, Brea, CA). Data were acquired and analyzed using CXP software (Beckman Coulter). The percentage of apoptotic cells were calculated based on the percentage of Annexin V and PI staining cells in HG or sample-treated groups *versus* the NG-treated group.

### Lactate dehydrogenase (LDH) release assay

HUVECs were seeded at a density of 5 × 10^4^ cells/well in 24-well plate and incubated with various concentrations of glucose (5.5, 15, 30 and 60 mM) in the presence or absence of ADC or RES for 24-72 h. After treatment, the amount of LDH in the HUVEC culture media was determined according to the manufacturer’s instruction. The LDH activity was measured at 440 nm using an ELISA microplate reader (Bio-Tek Instruments).

### Cell-cycle analyses

To determine the effect of ADC on the cell-cycle in HG-induced HUVECs, cells were grown in 6-well plates at a density of 1 × 10^5^ cells/well and treated with HG (30 mM) in the presence or absence of ADC (10 µM) or RES (5 µM) for 72 h. After treatment, cells were collected and then centrifuged for 3 min at 1500 × *g*. Cells were fixed with 95% cold ethanol and kept at -20°C overnight. The cell pellet was washed again with PBS and centrifuged at 1500 × *g* for 3 min, permeabilized with 1 mL PI/Triton X-100 (20 µg/mL PI, 0.1% Triton X-100 and 2.5 µg/mL RNAse) and incubated on ice for 30 min. The total cellular DNA content was analyzed with a flow cytometer (Beckman Coulter FC500) by acquiring at least 10,000 events. The analysis of the cell-cycle was performed by using CXP software (Beckman Coulter). The proliferation index (PI) of cells were calculated by the following formula as described previously [61]:

$$\mathrm{PI}\left(\%\right)=\frac{S+G_{2}/M}{G_{0}/G_{1}+S+G_{2}/M}\times 100$$

### SA-β-galactosidase activity assay

Senescence-associated β-galactosidase (SA-β-gal) activity was determined by using senescence associated β-galactosidase assay kit following the manufacturer’s procedure (Cell Signaling Technology, Danvers, CA). Briefly, HUVECs were grown in 6-well plates at a density of 5 × 10^4^ cells/well, and incubated with HG (30 mM) in the presence or absence or ADC (10 µM) or RES (5 µM) for 72 h. After incubation, cells were washed and fixed with 4% paraformaldehyde for 15 min, and stained with SA-β-gal staining solution at pH 6.0 overnight. The blue stained cells were observed and photographed under a bright-field microscope (Motic Electric Group, Xiamen, P.R. China). Number of SA-β-gal stained cells were counted/field and compared with NG. Results were expressed as the fold increase of SA-β-gal positive cells.

### Determination of intracellular ROS accumulation

Intracellular ROS accumulation in HUVECS was determined using fluorescent marker DCFH_2_-DA following a procedure described earlier with minor modifications [54]. Briefly, HUVECs (1 × 10^5^ cells/well) were seeded in 6-well plates and treated with HG (30 mM) in the presence or absence of ADC (10 µM) or RES (5 µM) for 1 hour. At the end of the incubation, the culture supernatant was removed and cells were washed twice with PBS. DCFH_2_-DA (10 µM) was mixed with 500 µL M199 medium and added to the culture plate. After incubation for 30 minutes, relative fluorescence intensity was measured using a fluorescence spectrophotometer (Hidex Oy) at 485/535 nm (A_485/535_). The percentage of ROS generation (%) was calculated as (A_485/535_ of treated cells/A_485/535_ of NG control cells) × 100.

### Immunofluorescence

Immunofluorescence analysis was performed as described previously [46]. Briefly, HUVECs at a density of 1 × 10^4^ cells/well were cultured in an eight-well glass Nunc Lab-Tek chamber (ThermoFisher Scientific, Waltham, MA), and treated with HG (30 mM) in the presence or absence of ADC (10 µM) or RES (5 µM) for 1 or 72 h. After treatment, culture medium was removed and cells were fixed in 4% paraformaldehyde for 15 min, permeabilized with 0.1% Triton X-100 for 10 min, washed and blocked with 10% FBS in PBS, and then incubated overnight with the corresponding primary antibodies (1:200) in 1.5% FBS. The cells were then incubated with the fluorescein isothiocyanate (FITC)-conjugated secondary antibody (1:1000) (Alexa fluor 488, ThermoFisher Scientific) for another 1 h in 6% bovine serum albumin (BSA). Then, the cells were stained with 1 μg/mL 4’,6-diamidino-2-phenylindole (DAPI, Cell Signaling Technology) for 5 min, washed with PBS, and visualized using a fluorescence microscope (Motic Electric Group) at 40 × magnification.

### Protein extraction and western blot analysis

Control and treated cells were lysed in either RIPA lysis buffer or nuclear and cytoplasmic extraction reagents (Thermo Fisher Scientific) following the manufacturer’s protocol. Protein content was determined by Bio-Rad protein assay reagent (Bio-Rad Laboratories, Hercules, CA). Extracted protein samples were denatured with 5% sample buffer by heating at 94°C for 5 min. Equal amounts of denatured protein samples (60 µg) were separated by 7-12% sodium dodecyl sulfate-poly acrylamide gel electrophoresis (SDS-PAGE) and the separated proteins were transferred onto polyvinylidene fluoride (PVDF) membrane overnight. The transferred protein membranes were incubated with 5% non-fat milk in Tris-buffered saline with Tween 20 (TBST) for 30 min at room temperature, followed by incubation with specific primary antibodies (1:1000) overnight, and inoculated with either horseradish peroxidase-conjugated goat anti-rabbit or anti-mouse antibodies (1:5000) for 2 h. The blots were developed with enhanced chemiluminescence (ECL) western blotting reagent (Millipore, Billerica, MA) and the luminescence signals were detected by using VL Chemi-Smart 3000 (Viogene Biotek, Sunnyvale, CA).

### RNA extraction and qPCR analysis

Total RNA was extracted from HUVECs using Trizol Reagent (Thermo Fisher Scientific) according to the manufacturer’s instructions. RNA concentration was quantified with a NanoVue Plus spectrophotometer (GE Health Care Life Sciences, Chicago, IL). An equal amount of total RNA (5 µg) was reverse-transcribed by using SuperScript III reverse transcriptase kit (Invitrogen). Quantification of mRNA expression for genes of interest was performed by qPCR using Applied Biosystems detection instruments and software (Applied Biosystems, Foster City, CA). qPCR reactions were performed with equal volume of cDNA, forward and reverse primers (10 µM), power SYBR Green master mix (Applied Biosystems) under the following conditions: 96°C for 3 minutes followed by 40 cycles at 96°C for 1 minute, 50°C for 30 seconds and 72°C for 90 seconds. GAPDH was used as an internal standard to control for variability in amplification because of differences in starting mRNA concentrations. The copy number of each transcript was calculated as the relative copy number normalized by GAPDH copy number. The primer sequences of each gene for qPCR were as follows. HO-1: forward primer (F), 5’-TCAACGGCACAGTCAAGG-3’; reverse primer (R), 5’-ACTCCACGACATACTCAGC-3’’ NQO-1: forward primer (F), 5’-TGCGGTGCAGCTCTTCTG-3’; reverse primer (R), 5’-GCAACCCGACAGCATGC-3’; GAPDH: forward primer (F), 5’-GATCATCAGCAATGCCTCCT-3’; reverse (R), 5’-TTCCTCTTGTGCTCTTGCTG-3’.

### Luciferase reporter assay

To determine the transcriptional activity of Nrf2, the antioxidant responsible element (ARE) promoter activity was determined by dual-luciferase reporter assay system (Promega, Madison, WI). Briefly, HUVECs (1 × 10^5^ cells/well) were seeded in 6-well plates, after achieving ∼80% confluence, cells were incubated in Opti-MEM for 5 h that did not contain antibiotics. Then, cells were transfected with ARE plasmid (Qiagen, Hilden, Germany) using Lipofectamine 2000 (Invitrogen) and further incubated for 36 h at 37°C. After plasmid transfection, cells were treated HG (30 mM) in the presence or absence of (10 µM) or RES (5 µM) for 6 h. Following incubation, the cells were lysed, incubated with luciferase agents and the relative luminescence intensity was quantified using a spectrophotometer (Hidex Oy, Turku, Finland).

### Gene silencing by siRNA

HUVECs (2.5 × 10^5^ cells/dish) were cultured in 6 cm dishes, after 60% confluence at the time of transfection, culture media was replaced with 2 mL of Opti-MEM (Invitrogen) and cells were transfected using Lipofectamine RNAiMax (Invitrogen) transfection reagent. For each transfection, 5 μL of RNAiMAX was mixed with 500 μL of Opti-MEM and incubated for 5 min at room temperature. In a separate tube, siRNA (100 pM for a final concentration of 100 nM in 1 mL Opti-MEM) was added to 500 μL of Opti-MEM and the siRNA solution was added to the diluted RNAiMAX reagent. The resulting siRNA/RNAiMAX mixture (1 mL) was incubated for an additional 25 min at room temperature to allow complex formation. Subsequently, the solution was added to the cells in the 6-well plates, giving a final transfection volume of 2 mL. After incubation for 6 h, the transfection medium was replaced with 3 mL of complete M199 medium and the cells were cultured at 37°C. After transfection for 24 h, cells were treated with HG (30 mM) in the presence or absence of ADC (10 µM) or RES (5 µM) or ZnPP (10 µM) and subjected to subsequent experiments.

### Statistical data analysis

Data are expressed as mean ± SD. All data were analyzed using the statistical software Graphpad Prism version 6.0 for Windows (GraphPad Software, La Jolla, CA). Statistical analysis was performed using one-way ANOVA followed by Dunnett’s multiple comparisons test with a *P* value of less than 0.05 indicating statistical significance.
